# {*N*′-[(2-Oxidonaphthalen-1-yl)methyl­idene]benzohydrazidato}(1,10-phenanthroline)copper(II) methanol monosolvate

**DOI:** 10.1107/S1600536811054316

**Published:** 2011-12-23

**Authors:** Bao-Lin Liu, De-Ming Dong, Xiu-Yi Hua, Jian-Wei Zhu

**Affiliations:** a College of Environment and Resources, Jilin University, Changchun 130012, People’s Republic of China; b College of Earth Sciences, Jilin University, Changchun 130061, People’s Republic of China

## Abstract

The title mononuclear complex, [Cu(C_18_H_12_N_2_O_2_)(C_12_H_8_N_2_)]·CH_3_OH, contains one *N*′-[(2-oxidonaphthalen-1-yl)methyl­idene]benzohydrazidate ligand (*L* ^2−^), a Cu^2+^ cation, one 1,10-phenanthroline ligand and a methanol solvent mol­ecule. The Cu^II^ ion adopts a CuO_2_N_3_ distorted square-pyramidal coordination. An O—H⋯O hydrogen bond is formed between the methanol solvent mol­ecule and the hydrazide O atom of the *L* ^2−^ ligand.

## Related literature

For details of the preparation of the Schiff base, see: Qiao *et al.* (2010 ▶). For applications of Schiff base compounds, see: Anford *et al.* (1998 ▶); Guo *et al.* (2010 ▶). For related structures, see: Huo *et al.* (2004 ▶); Liu *et al.* (2008 ▶); Sreeja *et al.* (2004 ▶).
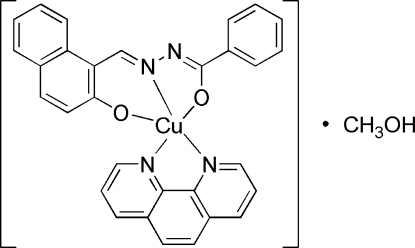


## Experimental

### 

#### Crystal data

[Cu(C_18_H_12_N_2_O_2_)(C_12_H_8_N_2_)]·CH_4_O
*M* *_r_* = 564.08
Monoclinic, 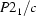

*a* = 20.388 (2) Å
*b* = 9.9707 (10) Å
*c* = 12.5268 (12) Å
β = 105.035 (2)°
*V* = 2459.4 (4) Å^3^
*Z* = 4
Mo *K*α radiation
μ = 0.93 mm^−1^
*T* = 185 K
0.20 × 0.18 × 0.17 mm

#### Data collection

Bruker APEXII CCD area-detector diffractometer
Absorption correction: multi-scan (*SADABS*; Bruker, 2007 ▶) *T*_min_ = 0.836, *T*_max_ = 0.858
12077 measured reflections
4352 independent reflections
2950 reflections with *I* > 2σ(*I*)
*R*_int_ = 0.059

#### Refinement

*R*[*F* ^2^ > 2σ(*F* ^2^)] = 0.042
*wR*(*F* ^2^) = 0.093
*S* = 1.02
4352 reflections
354 parameters
H-atom parameters constrained
Δρ_max_ = 0.34 e Å^−3^
Δρ_min_ = −0.32 e Å^−3^

### 

Data collection: *APEX2* (Bruker, 2007 ▶); cell refinement: *SAINT* (Bruker, 2007 ▶); data reduction: *SAINT*; program(s) used to solve structure: *SHELXS97* (Sheldrick, 2008 ▶); program(s) used to refine structure: *SHELXL97* (Sheldrick, 2008 ▶); molecular graphics: *DIAMOND* (Brandenburg, 1999 ▶); software used to prepare material for publication: *SHELXL97* and *publCIF* (Westrip, 2010) ▶.

## Supplementary Material

Crystal structure: contains datablock(s) I, global. DOI: 10.1107/S1600536811054316/kp2374sup1.cif

Structure factors: contains datablock(s) I. DOI: 10.1107/S1600536811054316/kp2374Isup2.hkl

Additional supplementary materials:  crystallographic information; 3D view; checkCIF report

## Figures and Tables

**Table 1:** Selected bond lengths (Å)

Cu1—O2	1.913 (2)
Cu1—N2	1.914 (2)
Cu1—O1	1.984 (2)
Cu1—N4	2.023 (3)
Cu1—N3	2.321 (3)

**Table 2:** Hydrogen-bond geometry (Å, °)

*D*—H⋯*A*	*D*—H	H⋯*A*	*D*⋯*A*	*D*—H⋯*A*
O3—H3*A*⋯O1	0.84	1.99	2.820 (3)	167

**Fig. 1.**
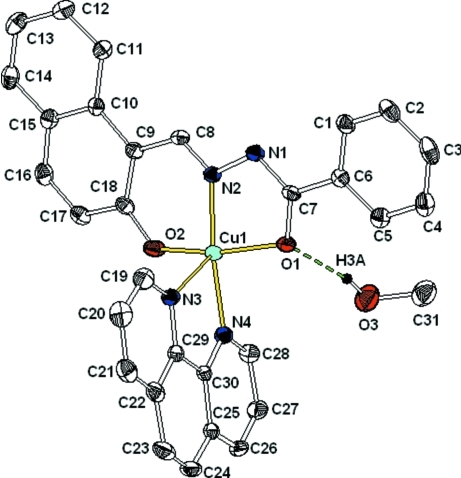
A view of the title organic compound, showing the atomic numbering scheme. Displacement ellipsoids are drawn at the 40% probability level.

## supplementary crystallographic information

### Comment

The study of transition metal-hydrazone compounds (Huo *et al.*, 2004), in which the acylhydrazone ligands are formed by condensing hydrazine with naphthaldehyde or their derivatives, has attracted considerable attention because of the magnetochemistry and chemical versatility of these compounds (Anford *et al.*, 1998; Guo *et al.*, 2010). We selected acylhydrazone ligand of 2-hydroxy-1-naphthaldehyde benzoylhydrazide (H_2_*L*) to construct coordination polymers with defined geometry. We report here the preparation and crystal structure of the title Schiff base copper(II) compound (Fig. 1, Table 1).  The title compound has a distorted square-pyramidal geometry and the central Cu^II^  is five-coordinated with the two O atoms and one N atom from H_2_*L* and two N atoms from 1,10-phenanthroline. Several mononuclear compounds with similar structures have been reported previously (Sreeja *et al.*, 2004; Liu *et al.*, 2008). The square plane around the Cu1 atom is formed by O_2_N_2_ donor atoms (O1, O2, N2 and N4 ). The apical position occupied by the second nitrogen atom (N3) of 1,10-phenanthroline with a larger distance than N4. The four basal atoms are coplanar showing a significant distortion from square geometry indicated by the trans-bond angle O1—Cu1—O2 [162.81 (9)°]. Cu^II^ is displaced from the basal plane in the direction of the axial nitrogen, which is evident from the bond angles of N2—Cu1—N4 [172.79 (11)°] and O1—Cu1—N2 [80.85 (10)°]. The maximum displacements from the least-squares plane through O1, O2, N2 and N4 are -0.0600 (21)Å and 0.1055 (27) Å for atoms O1 and N2, respectively; Cu1 is 0.2104 (4)Å below this plane. O3—H3A···O1 hydrogen bond is formed between the methanol solvent molecule and the O atom of the *L*^2-^ ligand (Table 2).

### Experimental

The 2-hydroxy-1-naphthaldehyde benzoylhydrazide ligand (H_2_*L*) was prepared in a similar manner to the reported procedures (Qiao *et al.* 2010). The title compound was synthesised by adding Cu(OAc)_2_.H_2_O (0.1 mmol) to a solution of H_2_*L* (0.1 mmol) and triethylamine (0.1 mmol) in methanol/dichloromethane (1:1 20 mL). After stirring for 3 h, 1,10-phenanthroline (0.1 mmol) was added to the resulting solution. Brown crystals of the title compound were isolated from the solution after two weeks.

### Refinement

H atoms bonded to C atoms were placed in calculated positions and refined using a riding model [C*sp^2^*–H = 0.95 Å; C*sp^3^*–H = 0.98 Å and *U*_ĩso_(H) = 1.2/1.5 *U*eq(C). H atoms bonded to methanol OH groups were located from difference Fourier series and then allowed to ride on their parent O atoms (AFIX 147) with *U*_ĩso_(H) = 1.2*U*eq(C) refined.

### Crystal data

**Table:**

[Cu(C_18_H_12_N_2_O_2_)(C_12_H_8_N_2_)]·CH_4_O	*F*(000) = 1164
*M**_r_* = 564.08	*D*_x_ = 1.523 Mg m^−^^3^
Monoclinic, *P*2_1_/*c*	Mo *K*α radiation, λ = 0.71073 Å
Hall symbol: -P 2ybc	Cell parameters from 3881 reflections
*a* = 20.388 (2) Å	θ = 2.3–25.1°
*b* = 9.9707 (10) Å	µ = 0.93 mm^−^^1^
*c* = 12.5268 (12) Å	*T* = 185 K
β = 105.035 (2)°	Block, brown
*V* = 2459.4 (4) Å^3^	0.20 × 0.18 × 0.17 mm
*Z* = 4	

### Data collection

**Table:**

Bruker APEXII CCD area-detector diffractometer	4352 independent reflections
Radiation source: fine-focus sealed tube	2950 reflections with *I* > 2σ(*I*)
graphite	*R*_int_ = 0.059
phi and ω scans	θ_max_ = 25.1°, θ_min_ = 2.1°
Absorption correction: multi-scan (*SADABS*; Bruker, 2007)	*h* = −21→24
*T*_min_ = 0.836, *T*_max_ = 0.858	*k* = −11→10
12077 measured reflections	*l* = −14→12

### Refinement

**Table:**

Refinement on *F*^2^	Primary atom site location: structure-invariant direct methods
Least-squares matrix: full	Secondary atom site location: difference Fourier map
*R*[*F*^2^ > 2σ(*F*^2^)] = 0.042	Hydrogen site location: inferred from neighbouring sites
*wR*(*F*^2^) = 0.093	H-atom parameters constrained
*S* = 1.02	*w* = 1/[σ^2^(*F*_o_^2^) + (0.0369*P*)^2^ + 0.1098*P*] where *P* = (*F*_o_^2^ + 2*F*_c_^2^)/3
4352 reflections	(Δ/σ)_max_ < 0.001
354 parameters	Δρ_max_ = 0.34 e Å^−^^3^
0 restraints	Δρ_min_ = −0.32 e Å^−^^3^

### Special details

**Table:**

Geometry. All esds (except the esd in the dihedral angle between two l.s. planes) are estimated using the full covariance matrix. The cell esds are taken into account individually in the estimation of esds in distances, angles and torsion angles; correlations between esds in cell parameters are only used when they are defined by crystal symmetry. An approximate (isotropic) treatment of cell esds is used for estimating esds involving l.s. planes.
Refinement. Refinement of F^2^ against ALL reflections. The weighted R-factor wR and goodness of fit S are based on F^2^, conventional R-factors R are based on F, with F set to zero for negative F^2^. The threshold expression of F^2^ > 2sigma(F^2^) is used only for calculating R-factors(gt) etc. and is not relevant to the choice of reflections for refinement. R-factors based on F^2^ are statistically about twice as large as those based on F, and R- factors based on ALL data will be even larger.

### Fractional atomic coordinates and isotropic or equivalent isotropic displacement parameters (Å^2^)

**Table:**

	*x*	*y*	*z*	*U*_iso_*/*U*_eq_	
Cu1	0.25702 (2)	0.62657 (4)	0.14434 (3)	0.02321 (13)	
N2	0.22403 (12)	0.6138 (3)	0.2738 (2)	0.0210 (6)	
N4	0.30304 (13)	0.6479 (3)	0.0204 (2)	0.0243 (7)	
O1	0.32626 (11)	0.5026 (2)	0.23282 (18)	0.0248 (5)	
O2	0.20269 (11)	0.7819 (2)	0.09414 (18)	0.0272 (5)	
N1	0.25609 (13)	0.5187 (3)	0.3516 (2)	0.0225 (6)	
C22	0.21141 (17)	0.3799 (3)	−0.1607 (3)	0.0281 (8)	
C10	0.08817 (15)	0.8615 (3)	0.2718 (3)	0.0201 (7)	
C30	0.28412 (16)	0.5641 (3)	−0.0679 (3)	0.0219 (8)	
C18	0.15699 (16)	0.8359 (3)	0.1370 (3)	0.0230 (8)	
C17	0.12057 (16)	0.9477 (3)	0.0781 (3)	0.0283 (8)	
H17	0.1332	0.9819	0.0155	0.034*	
C19	0.15462 (17)	0.3786 (4)	0.0142 (3)	0.0334 (9)	
H19	0.1345	0.3769	0.0747	0.040*	
C29	0.23126 (16)	0.4680 (3)	−0.0704 (3)	0.0223 (8)	
N3	0.20374 (13)	0.4662 (3)	0.0168 (2)	0.0237 (7)	
C28	0.35071 (17)	0.7391 (3)	0.0217 (3)	0.0298 (8)	
H28	0.3632	0.7984	0.0830	0.036*	
C7	0.30846 (16)	0.4681 (3)	0.3224 (3)	0.0218 (8)	
C6	0.34865 (15)	0.3646 (3)	0.3957 (3)	0.0224 (7)	
C5	0.40428 (16)	0.3049 (3)	0.3705 (3)	0.0283 (8)	
H5	0.4179	0.3337	0.3073	0.034*	
C8	0.17973 (15)	0.6886 (3)	0.3031 (3)	0.0211 (7)	
H8	0.1713	0.6720	0.3730	0.025*	
C15	0.04909 (16)	0.9624 (3)	0.2047 (3)	0.0253 (8)	
C27	0.38318 (17)	0.7519 (4)	−0.0626 (3)	0.0327 (9)	
H27	0.4168	0.8190	−0.0590	0.039*	
C25	0.31509 (16)	0.5700 (3)	−0.1560 (3)	0.0252 (8)	
C9	0.14253 (16)	0.7951 (3)	0.2367 (3)	0.0213 (7)	
C24	0.29397 (18)	0.4778 (4)	−0.2454 (3)	0.0337 (9)	
H24	0.3152	0.4805	−0.3046	0.040*	
C16	0.06880 (17)	1.0053 (3)	0.1098 (3)	0.0294 (8)	
H16	0.0448	1.0770	0.0670	0.035*	
C4	0.44030 (18)	0.2036 (3)	0.4365 (3)	0.0346 (9)	
H4	0.4786	0.1642	0.4187	0.042*	
C23	0.24477 (18)	0.3873 (4)	−0.2479 (3)	0.0343 (9)	
H23	0.2319	0.3271	−0.3086	0.041*	
C12	0.01612 (16)	0.8884 (3)	0.3977 (3)	0.0322 (9)	
H12	0.0051	0.8635	0.4642	0.039*	
C20	0.13060 (19)	0.2883 (3)	−0.0727 (3)	0.0377 (10)	
H20	0.0950	0.2275	−0.0711	0.045*	
C21	0.15930 (17)	0.2894 (3)	−0.1600 (3)	0.0352 (9)	
H21	0.1438	0.2289	−0.2198	0.042*	
C13	−0.02362 (18)	0.9835 (3)	0.3284 (3)	0.0353 (9)	
H13	−0.0619	1.0222	0.3466	0.042*	
C11	0.07096 (16)	0.8301 (3)	0.3714 (3)	0.0251 (8)	
H11	0.0979	0.7675	0.4211	0.030*	
C2	0.36495 (18)	0.2189 (3)	0.5537 (3)	0.0336 (9)	
H2	0.3510	0.1889	0.6164	0.040*	
C1	0.32968 (17)	0.3206 (3)	0.4892 (3)	0.0274 (8)	
H1	0.2922	0.3612	0.5084	0.033*	
C14	−0.00667 (17)	1.0199 (3)	0.2342 (3)	0.0331 (9)	
H14	−0.0332	1.0856	0.1873	0.040*	
C26	0.36606 (17)	0.6671 (3)	−0.1501 (3)	0.0300 (9)	
H26	0.3885	0.6732	−0.2076	0.036*	
C3	0.42037 (19)	0.1600 (3)	0.5282 (3)	0.0381 (10)	
H3	0.4446	0.0903	0.5733	0.046*	
O3	0.45503 (12)	0.6073 (3)	0.2347 (2)	0.0496 (7)	
H3A	0.4200	0.5690	0.2430	0.074*	
C31	0.51281 (18)	0.5443 (4)	0.3014 (4)	0.0487 (11)	
H31A	0.5537	0.5877	0.2906	0.073*	
H31B	0.5128	0.4494	0.2810	0.073*	
H31C	0.5122	0.5518	0.3792	0.073*	

### Atomic displacement parameters (Å^2^)

**Table:**

	*U*^11^	*U*^22^	*U*^33^	*U*^12^	*U*^13^	*U*^23^
Cu1	0.0281 (2)	0.0251 (2)	0.0184 (2)	0.0030 (2)	0.00950 (17)	0.0013 (2)
N2	0.0245 (14)	0.0205 (15)	0.0193 (15)	0.0015 (13)	0.0081 (12)	0.0036 (13)
N4	0.0260 (15)	0.0254 (16)	0.0228 (17)	0.0045 (13)	0.0087 (13)	0.0020 (13)
O1	0.0274 (12)	0.0288 (13)	0.0199 (14)	0.0057 (10)	0.0090 (11)	0.0031 (10)
O2	0.0360 (13)	0.0264 (13)	0.0220 (14)	0.0046 (11)	0.0124 (11)	0.0042 (10)
N1	0.0271 (16)	0.0227 (15)	0.0187 (16)	0.0027 (12)	0.0079 (13)	0.0015 (13)
C22	0.0337 (19)	0.0243 (19)	0.024 (2)	0.0075 (18)	0.0038 (16)	0.0030 (17)
C10	0.0194 (16)	0.0169 (17)	0.0224 (19)	−0.0027 (14)	0.0026 (14)	−0.0041 (15)
C30	0.0228 (18)	0.0262 (19)	0.0174 (19)	0.0095 (15)	0.0065 (16)	0.0066 (15)
C18	0.0289 (19)	0.0201 (19)	0.0185 (19)	−0.0016 (14)	0.0032 (16)	−0.0031 (14)
C17	0.036 (2)	0.026 (2)	0.022 (2)	−0.0012 (16)	0.0056 (18)	0.0032 (16)
C19	0.034 (2)	0.031 (2)	0.036 (2)	0.0011 (19)	0.0110 (18)	0.0088 (19)
C29	0.0224 (18)	0.0227 (19)	0.022 (2)	0.0086 (15)	0.0055 (16)	0.0038 (15)
N3	0.0268 (16)	0.0256 (16)	0.0210 (17)	0.0045 (13)	0.0104 (14)	0.0041 (12)
C28	0.032 (2)	0.033 (2)	0.026 (2)	0.0006 (17)	0.0095 (17)	0.0021 (17)
C7	0.0259 (19)	0.0244 (19)	0.0153 (19)	−0.0037 (15)	0.0059 (16)	−0.0025 (14)
C6	0.0232 (17)	0.0203 (17)	0.0228 (19)	−0.0037 (16)	0.0041 (15)	−0.0006 (16)
C5	0.0295 (19)	0.0247 (19)	0.031 (2)	0.0005 (16)	0.0073 (17)	−0.0022 (16)
C8	0.0247 (18)	0.0218 (18)	0.0187 (19)	−0.0035 (15)	0.0089 (16)	−0.0026 (15)
C15	0.0241 (19)	0.0218 (19)	0.030 (2)	0.0010 (15)	0.0062 (17)	−0.0016 (16)
C27	0.029 (2)	0.040 (2)	0.032 (2)	0.0010 (17)	0.0136 (18)	0.0117 (18)
C25	0.0258 (19)	0.0297 (19)	0.021 (2)	0.0111 (16)	0.0067 (17)	0.0051 (16)
C9	0.0244 (18)	0.0197 (18)	0.0193 (19)	−0.0012 (15)	0.0050 (15)	−0.0006 (15)
C24	0.042 (2)	0.041 (2)	0.022 (2)	0.0164 (19)	0.0154 (19)	0.0033 (17)
C16	0.0294 (19)	0.0234 (19)	0.032 (2)	0.0071 (16)	0.0024 (18)	0.0030 (16)
C4	0.032 (2)	0.030 (2)	0.037 (2)	0.0038 (17)	0.0012 (19)	−0.0067 (18)
C23	0.047 (2)	0.034 (2)	0.021 (2)	0.008 (2)	0.0072 (18)	−0.0025 (18)
C12	0.032 (2)	0.033 (2)	0.036 (2)	−0.0023 (18)	0.0159 (18)	−0.0052 (18)
C20	0.040 (2)	0.026 (2)	0.045 (3)	−0.0039 (18)	0.007 (2)	0.0040 (19)
C21	0.039 (2)	0.028 (2)	0.033 (2)	0.0024 (18)	0.0009 (19)	−0.0030 (17)
C13	0.029 (2)	0.032 (2)	0.048 (3)	0.0073 (18)	0.016 (2)	−0.0036 (19)
C11	0.0256 (18)	0.0216 (19)	0.029 (2)	0.0011 (15)	0.0090 (17)	0.0004 (15)
C2	0.037 (2)	0.033 (2)	0.026 (2)	−0.0103 (18)	−0.0001 (18)	0.0073 (17)
C1	0.0255 (19)	0.032 (2)	0.023 (2)	−0.0059 (16)	0.0034 (17)	−0.0017 (16)
C14	0.028 (2)	0.027 (2)	0.041 (3)	0.0077 (16)	0.0046 (19)	0.0043 (17)
C26	0.029 (2)	0.040 (2)	0.025 (2)	0.0100 (17)	0.0146 (18)	0.0105 (17)
C3	0.041 (2)	0.027 (2)	0.037 (2)	0.0005 (17)	−0.008 (2)	0.0085 (17)
O3	0.0353 (15)	0.0576 (19)	0.057 (2)	−0.0004 (15)	0.0143 (15)	0.0074 (15)
C31	0.035 (2)	0.056 (3)	0.056 (3)	−0.007 (2)	0.014 (2)	−0.009 (2)

### Geometric parameters (Å, °)

**Table:**

Cu1—O2	1.913 (2)	C8—C9	1.437 (4)
Cu1—N2	1.914 (2)	C8—H8	0.9500
Cu1—O1	1.984 (2)	C15—C14	1.405 (4)
Cu1—N4	2.023 (3)	C15—C16	1.417 (5)
Cu1—N3	2.321 (3)	C27—C26	1.357 (5)
N2—C8	1.296 (4)	C27—H27	0.9500
N2—N1	1.396 (3)	C25—C26	1.408 (4)
N4—C28	1.328 (4)	C25—C24	1.427 (5)
N4—C30	1.360 (4)	C24—C23	1.344 (4)
O1—C7	1.312 (4)	C24—H24	0.9500
O2—C18	1.306 (3)	C16—H16	0.9500
N1—C7	1.315 (4)	C4—C3	1.384 (5)
C22—C21	1.395 (4)	C4—H4	0.9500
C22—C29	1.406 (4)	C23—H23	0.9500
C22—C23	1.431 (4)	C12—C11	1.374 (4)
C10—C11	1.416 (4)	C12—C13	1.395 (5)
C10—C15	1.416 (4)	C12—H12	0.9500
C10—C9	1.454 (4)	C20—C21	1.367 (5)
C30—C25	1.407 (4)	C20—H20	0.9500
C30—C29	1.436 (4)	C21—H21	0.9500
C18—C9	1.416 (4)	C13—C14	1.362 (5)
C18—C17	1.433 (4)	C13—H13	0.9500
C17—C16	1.349 (4)	C11—H11	0.9500
C17—H17	0.9500	C2—C1	1.378 (4)
C19—N3	1.323 (4)	C2—C3	1.382 (5)
C19—C20	1.400 (5)	C2—H2	0.9500
C19—H19	0.9500	C1—H1	0.9500
C29—N3	1.352 (4)	C14—H14	0.9500
C28—C27	1.390 (4)	C26—H26	0.9500
C28—H28	0.9500	C3—H3	0.9500
C7—C6	1.480 (4)	O3—C31	1.403 (4)
C6—C5	1.387 (4)	O3—H3A	0.8400
C6—C1	1.396 (4)	C31—H31A	0.9800
C5—C4	1.388 (5)	C31—H31B	0.9800
C5—H5	0.9500	C31—H31C	0.9800
			
O2—Cu1—N2	91.80 (10)	C10—C15—C16	118.3 (3)
O2—Cu1—O1	162.81 (9)	C26—C27—C28	118.9 (3)
N2—Cu1—O1	80.85 (10)	C26—C27—H27	120.6
O2—Cu1—N4	90.39 (10)	C28—C27—H27	120.6
N2—Cu1—N4	172.79 (11)	C30—C25—C26	117.6 (3)
O1—Cu1—N4	95.16 (9)	C30—C25—C24	118.9 (3)
O2—Cu1—N3	101.75 (9)	C26—C25—C24	123.5 (3)
N2—Cu1—N3	109.43 (10)	C18—C9—C8	121.6 (3)
O1—Cu1—N3	95.36 (9)	C18—C9—C10	119.0 (3)
N4—Cu1—N3	76.80 (10)	C8—C9—C10	119.4 (3)
C8—N2—N1	115.4 (3)	C23—C24—C25	121.5 (3)
C8—N2—Cu1	128.7 (2)	C23—C24—H24	119.3
N1—N2—Cu1	115.65 (18)	C25—C24—H24	119.3
C28—N4—C30	118.9 (3)	C17—C16—C15	122.2 (3)
C28—N4—Cu1	123.1 (2)	C17—C16—H16	118.9
C30—N4—Cu1	118.0 (2)	C15—C16—H16	118.9
C7—O1—Cu1	109.05 (19)	C3—C4—C5	119.9 (3)
C18—O2—Cu1	127.8 (2)	C3—C4—H4	120.1
C7—N1—N2	109.5 (2)	C5—C4—H4	120.1
C21—C22—C29	117.3 (3)	C24—C23—C22	121.1 (3)
C21—C22—C23	123.5 (3)	C24—C23—H23	119.4
C29—C22—C23	119.2 (3)	C22—C23—H23	119.4
C11—C10—C15	116.7 (3)	C11—C12—C13	121.0 (3)
C11—C10—C9	123.3 (3)	C11—C12—H12	119.5
C15—C10—C9	120.0 (3)	C13—C12—H12	119.5
N4—C30—C25	121.4 (3)	C21—C20—C19	118.8 (3)
N4—C30—C29	118.7 (3)	C21—C20—H20	120.6
C25—C30—C29	119.9 (3)	C19—C20—H20	120.6
O2—C18—C9	125.3 (3)	C20—C21—C22	119.7 (3)
O2—C18—C17	116.0 (3)	C20—C21—H21	120.2
C9—C18—C17	118.6 (3)	C22—C21—H21	120.2
C16—C17—C18	121.4 (3)	C14—C13—C12	118.9 (3)
C16—C17—H17	119.3	C14—C13—H13	120.5
C18—C17—H17	119.3	C12—C13—H13	120.5
N3—C19—C20	123.6 (3)	C12—C11—C10	121.4 (3)
N3—C19—H19	118.2	C12—C11—H11	119.3
C20—C19—H19	118.2	C10—C11—H11	119.3
N3—C29—C22	123.3 (3)	C1—C2—C3	120.7 (3)
N3—C29—C30	117.2 (3)	C1—C2—H2	119.7
C22—C29—C30	119.5 (3)	C3—C2—H2	119.7
C19—N3—C29	117.3 (3)	C2—C1—C6	120.5 (3)
C19—N3—Cu1	133.5 (2)	C2—C1—H1	119.8
C29—N3—Cu1	109.2 (2)	C6—C1—H1	119.8
N4—C28—C27	123.0 (3)	C13—C14—C15	121.6 (3)
N4—C28—H28	118.5	C13—C14—H14	119.2
C27—C28—H28	118.5	C15—C14—H14	119.2
O1—C7—N1	124.2 (3)	C27—C26—C25	120.2 (3)
O1—C7—C6	118.8 (3)	C27—C26—H26	119.9
N1—C7—C6	117.0 (3)	C25—C26—H26	119.9
C5—C6—C1	118.5 (3)	C2—C3—C4	119.5 (3)
C5—C6—C7	120.8 (3)	C2—C3—H3	120.2
C1—C6—C7	120.7 (3)	C4—C3—H3	120.2
C6—C5—C4	121.0 (3)	C31—O3—H3A	109.5
C6—C5—H5	119.5	O3—C31—H31A	109.5
C4—C5—H5	119.5	O3—C31—H31B	109.5
N2—C8—C9	124.3 (3)	H31A—C31—H31B	109.5
N2—C8—H8	117.9	O3—C31—H31C	109.5
C9—C8—H8	117.9	H31A—C31—H31C	109.5
C14—C15—C10	120.2 (3)	H31B—C31—H31C	109.5
C14—C15—C16	121.5 (3)		

### Hydrogen-bond geometry (Å, °)

**Table:**

*D*—H···*A*	*D*—H	H···*A*	*D*···*A*	*D*—H···*A*
O3—H3A···O1	0.84	1.99	2.820 (3)	167.
